# LCTX-F2, a Novel Potentiator of Coagulation Factors From the Spider Venom of *Lycosa singoriensis*

**DOI:** 10.3389/fphar.2020.00896

**Published:** 2020-06-16

**Authors:** Pengpeng Li, Zhongzhe Zhang, Qiong Liao, Er Meng, James Mwangi, Ren Lai, Mingqiang Rong

**Affiliations:** ^1^The National & Local Joint Engineering Laboratory of Animal Peptide Drug Development, College of Life Sciences, Hunan Normal University, Changsha, China; ^2^School of Life Sciences, Hunan University of Science and Technology, Xiangtan, China; ^3^Key Laboratory of Animal Models and Human Disease Mechanisms of Chinese Academy of Sciences & Yunnan Province, Kunming Institute of Zoology, Kunming, China

**Keywords:** spider venom, peptide, coagulant, coagulation factors, toxin

## Abstract

Spider venoms contain many functional proteins/peptides such as proteinases, serine/cysteine proteinase inhibitors, insecticidal toxins, and ion channel toxins. However, to date, no peptide toxin with procoagulant activities has been identified from spider venom. In this study, a novel toxin LCTX-F2 with coagulation-promoting activity was identified and characterized in the venom of the spider *Lycosa singoriensis* (*L. singoriensis*). LCTX-F2 significantly shortened activated partial thromboplastin time (APTT), clotting time, and plasma recalcification time. This toxin directly interacted with several coagulation factors such as FXIIa, kallikrein, thrombin, and FXa and increased their protease activities. In liver bleeding and tail bleeding mouse models, LCTX-F2 significantly decreased the number of blood cells and bleeding time in a dose-dependent manner. At the same dosage, LCTX-F2 exhibited a more significant procoagulant effect than epsilon aminocaproic acid (EACA). Moreover, LCTX-F2 showed no cytotoxic or hemolytic activity against either normal cells or red blood cells. Our results suggested that LCTX-F2 is a potentiator of coagulation factors with the potential for use in the development of procoagulant drugs.

## Introduction

Excessive blood loss is a leading cause of the high mortality rate associated with surgical operations (Willner et al., 2016). Reducing blood loss and managing hemostasis are vital during surgery and hemorrhagic shock (Zakaria et al., 2005). In surgical operations, tranexamic acid (TXA), epsilon aminocaproic acid (EACA) (Alkjaersig et al., 1959), aprotinin, and recombinant activated factor VII (rFVIIa) are the most commonly used procoagulant drugs. The two antifibrinolytic agents, TXA and EACA, competitively inhibit the conversion of plasminogen to plasmin. Meanwhile, aprotinin is used to reduce bleeding during complex heart and liver surgery, and its main function is to slow down fibrinolysis so as to allow the breakdown of blood clots (Willner et al., 2016).

Several proteins that promote coagulation have been isolated from the venom of the snake *Pseudonaja textilis*. a factor Xa-like protein, Haempatch™, which exhibits potent procoagulant effects, has been developed as a haemostatic drug, and is used to reduce blood loss due to surgery or trauma. CoVase™ is regarded as a procoagulant cofactor. It is a systemic antibleeding medication used to treat noncompressible hemorrhage and internal bleeding (Fry et al., 2005). Textilinin-1, a selective plasmin inhibitor, can reduce blood loss associated with complex surgeries (Flight et al., 2009). Those procoagulant proteins affect multiple steps in the hemostatic process, ranging from activation of coagulation factors to platelet aggregation (Santos et al., 2000).

Abundant evidence has demonstrated that spider venoms contain many active peptide components (Meng et al., 2016). Most peptides identified in spider venom are small, disulfide-rich neurotoxins. Spiders of the genus *Loxosceles* (Tavares et al., 2011; Tavares et al., 2016), also known as brown spiders, are widely distributed in South America and their venom can cause a necrotizing-hemolytic syndrome in humans, as well as hemorrhagic problems, hemolysis, platelet aggregation, and renal failure (Tavares et al., 2004; Van Den Berg et al., 2007; Nguyen and Pandey, 2019); however, the mechanism underlying these venom-induced hemorrhagic disorders is poorly understood. *Lycosa singoriensis* (*L. singoriensis*) is distributed throughout the west of China and its venom causes severe illness, including pain, cutaneous necrosis, nausea, vomiting, and even dyspnea. In this study, a novel peptide with procoagulant activity, LCTX-F2, was identified in the venom of *L. singoriensis* and characterized. LCTX-F2 significantly shortened activated partial thromboplastin time (APTT), clotting time, and plasma recalcification time. We further employed *in vivo* mouse models of tail bleeding and liver bleeding to investigate the mechanism underlying the procoagulant activity of LCTX-F2.

## Materials and Methods

### Animals and Cells

The BALB/c mice used in this experiment were purchased from Kunming Medical University. HEK-293T (Human embryonic kidney 293T) and CHO (Chinese hamster ovary 1) cells were purchased from the cell bank of the Kunming Institute of Zoology. All experiments were performed in accordance with national legislation and approved by the animal experimental ethics committee (SMKX-20170210) of the Kunming Institute of Zoology, Chinese Academy of Sciences.

### cDNA Library Construction and Sequencing

The *L. singoriensis* sample was stored at the Kunming Museum of Zoology (Kunming City, Yunnan Province, China). The venom gland of *L. singoriensis* was dissected and used for the construction of a cDNA library (Yang et al., 2012). Total RNA was extracted from the venom glands of 20 spiders using TRIzol reagent (Life Technologies Ltd, Carlsbad, CA, USA) following the manufacturer’s instructions. The cDNA library was constructed using the Creator SMART cDNA Library Construction Kit (Clontech, Palo Alto, CA, USA) according to manufacturer’s instructions. A cDNA library with 3 × 10^6^ independent colonies was produced. All the clones from the cDNA library were sequenced on an ABI PRISM 377 DNA sequencer (Applied Biosystems, Foster City, CA, USA). cDNA fragments longer than 200 bp were selected for further analyses. The LCTX-F2 sequence has been uploaded to GenBank of NCBI under the accession number BankIt 2321489 MT178457.

### Expression and Purification of Recombinant LCTX-F2

The DNA sequence of LCTX-F2 was synthesized (Sangon Biotech, Co., Ltd, Shanghai, China) and a TEV enzyme cleavage site (ENLYFQG) was inserted immediately upstream of the sequence (Ma et al., 2016). For recombinant expression, an LCTX-F2/pET32a (+) prokaryotic expression vector was transformed into *Escherichia coli* strain BL-21 (DE3). LCTX-F2 was cleaved from the recombinant protein using the TEV enzyme (Beijing Solarbio Science & Technology Co., Ltd). The hydrolyzed LCTX-F2 was first separated by molecular exclusion using a Sephadex G-75 gel filtration column (2.6 cm × 100 cm; GE Healthcare Bio-Sciences AB, Uppsala, Sweden) with phosphate-buffered saline (PBS) (150 mM NaCl, 2.7 mM KCl, 1.5 mM KH_2_PO_4_, and 8 mM K_2_HPO_4_, pH 7.2). Then, LCTX-F2 was further eluted by reversed-phase high-performance liquid chromatography (RP-HPLC) using a C18 column (Unisil C18 column, 5-μm particle size, 10 × 250 mm). The gradient of high-performance liquid chromatography solution (0.1% trifluoroacetic acid/acetyl) increased by 1% per min. LCTX-F2 was dissolved in 0.1% trifluoroacetic acid/water (0.5 μl) and spotted onto a plate with 0.5-μl matrix (10 mg/ml α-cyano-4-hydroxycinnamic acid in 60% acetonitrile). The molecular weight of LCTX-F2 was determined using an UltraFlex I mass spectrometer (UltraFlex I, Bruker Daltonics, Billerica, MA, USA) in positive ion mode. The accuracy of mass determination was recorded according to the manufacturer’s instructions. The purity of LCTX-F2 was approximately 95%.

### Blood Coagulation Time Assay

Blood recalcification time was measured according to a previously described method (Gulliani et al., 1976). The platelet-poor plasma (PPP) negative control obtained from healthy human subjects was transferred to 96-well plates. LCTX-F2 (final concentration ranging from 0.0 to 3.0 μM) was dissolved in 80 μl of HEPES buffer (10 mM HEPES, 150 mM NaCl, pH 7.4) and added to the plates with 20 μl of PPP. Two samples were incubated for 10 min at room temperature. Before recording clotting time, 50 μl of CaCl_2_ (25 mM) was dispensed into the 96-well plates. The APTT was assayed as previously described (Jung and Kim, 2009). APTT reagent (50 μl), PPP (40 μl), and LCTX-F2 (10 μl) were incubated for 5 min at 37°C. The APTT was recorded after the addition of 50 μl of CaCl_2_ (25 mM). The prothrombin time (PT) was assayed. PPP (40 μl) and LCTX-F2 (10 μl) were incubated for 5 min at 37°C. The PT was recorded after the addition of 50 μl of PT reagent. Three parallel tests were performed and the absorbance was recorded at 650 nm using a microplate spectrophotometer (BioTek Instrument, Inc., Winooski, VT, USA).

### Determination of Serine Protease Activity

Serine protease activity was tested in Tris–HCl buffer (0.05 M, pH 7.8) at 37°C (Ma et al., 2016). The proteases factor Xa (HFXa 1011), trypsin (T4665), thrombin (T4393), plasmin (P1867), factor XIIa (HFXIIa1212a), and kallikrein (HPKa 1303) were preincubated with different doses of LCTX-F2 (0.0–3.0 μM) for 10 min at 37°C. Factor Xa, factor XIIa, and kallikrein were purchased from Enzyme Research Laboratories (South Bend, IN, USA). Thrombin, trypsin, and plasmin were purchased from Sigma (St. Louis, MO, USA). LCTX-F2 was mixed with the enzymes for 10 min, after which 0.5 mM of substrate was added to initiate each assay. The substrate for FXa, trypsin, thrombin, plasmin, and kallikrein/factor XIIa were F3301, B3133, H-D-Phe-Pip-Arg-pNa·2HCl, G8148, and H-D-Pro-Phe-ArgpNA·2HCl, respectively. All the substrates were purchased from Sigma, except H-D-Phe-Pip-Arg-pNa·2HCl and G8148 which were purchased from Hyphen Biomed (Neuville-sur-Oise, France). The LCTX-F2 enzyme reactions were continuously measured for 20 min at 405 nm.

### Surface Plasmon Resonance Assay

The binding of LCTX-F2 to FXIIa, kallikrein, FXa, and thrombin was tested with a Biacore 3000 (GE Healthcare Bio-Sciences, Uppsala, Sweden) according to a previously described method (Konings et al., 2011; Zhang et al., 2012). The experiments were performed at 25°C using HBS-EP buffer as a running buffer (10 mM HEPES, pH 7.4, 3 mM EDTA, 150 mM NaCl, and 0.05% P20 surfactant). LCTX-F2 as a ligand was diluted with running buffer and coupled to CM5 by amine coupling, yielding a total of 2,000 response units. The running buffer flow rate was maintained at 30 μl/min. A series of twofold dilutions of FXIIa, kallikrein, FXa, and thrombin, as analytes, were generated using running buffer (0, 3.125, 6.25, 12.5, 25, 50, 100 μg/ml). Solutions of the different analytes were injected over the flow channel at a flow rate of 30 μl/min for 120 s, followed by dissociation for 300 s. The LCTX-F2/enzyme binding curve was acquired by subtracting the FL1 curve from the FL2 curve. All surface plasmon resonance (SPR) experiments were performed in triplicate and analyzed by BIA evaluation software.

### Tail Bleeding Assay

Six BALB/c mice (20–25 g) were used in each group. The mice were anesthetized by intraperitoneal injection of 100 μl of a 0.3% pentobarbital sodium solution. Subsequently, saline, LCTX-F2 (0.625, 1.25, or 2.5 mg/kg), or EACA (5 mg/kg) was separately injected into the tail vein. After 10 min, a 7-mm tail-tip transection was made according to a previously described method (Schoenwaelder et al., 2011) to estimate the hemostatic effect. After transection, the bleeding tail was quickly steeped in saline solution at 37°C and the timing began. Bleeding time was recorded from the beginning of tail transection until 120 s after blood flow had stopped.

### Liver Bleeding Assay

Seven BALB/c mice (20–25 g) were used in each group. The mice were anesthetized by intraperitoneal injection of 100 μl of a 0.3% pentobarbital sodium solution. Subsequently, saline, LCTX-F2 (1.25, 2.5, or 5 mg/kg), or EACA (5 mg/kg) were separately injected into the tail vein. After 10 min, scissors were used to open the thoracic cavity along the ventral white line. A piece (5.00 ± 0.15 mg) was cut from the lower edge of the right liver lobe (Tilly et al., 2014) and the thoracic cavity closed. After 20 min, the number of RBCs was calculated after peritoneal lavage with 4 ml of a 0.9% saline solution.

### Cytotoxicity Assay

A cell counting kit-8 (CCK-8, HY-K0301; MedChemExpress, Monmouth Junction, NJ, USA) was used to evaluate the effect of LCTX-F2 on cell viability (Wang et al., 2010). HEK-293T and CHO cells were incubated with LCTX-F2 (0, 5, 10, 20, or 100 mg/ml) for 24 h. The cells were subsequently incubated with 0.2 ml of culture medium containing 10% CCK-8 reagent for 2 h. The absorbance was monitored at 450 nm using a microplate spectrophotometer.

### Hemolysis Assay

A rabbit RBC suspension (2%) in PBS was obtained for subsequent experiments as previously described (Vives et al., 1997). Rabbit RBC suspensions were incubated with PBS, LCTX-F2 (5, 10, 20, or 100 mg/ml), or distilled water as positive control for 3 h at room temperature. After centrifugation at 10,050 rpm for 3 min, the absorbance of the supernatants was measured spectrophotometrically at 570 nm to calculate the hemolysis ratio.

### Statistical Analysis

Data are presented as means ± SD from three independent experiments. Significance was determined by unpaired *t*-test; *P* < 0.05 was considered significant.

## Results

### Identification of LCTX-F2

**Figure 1:**
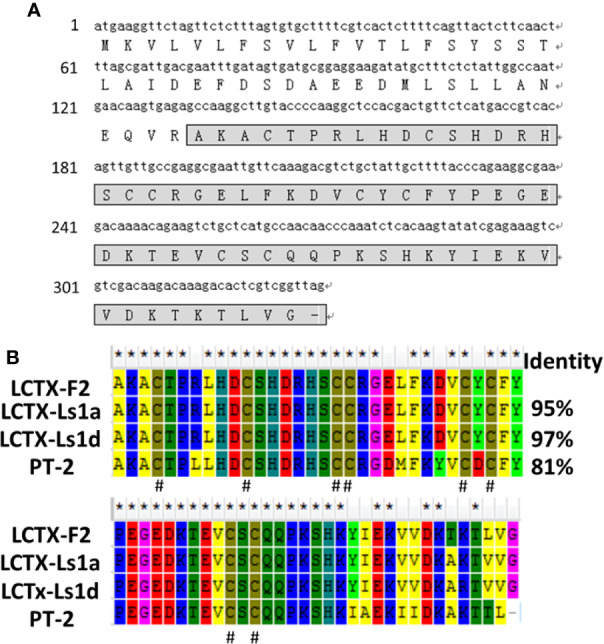
Amino acid and cDNA sequence of LCTX-F2. **(A)** The cDNA sequence of LCTX-F2. The sequence of mature LCTX-F2 is boxed and the stop codon is indicated by a bar (–). **(B)** NCBI-BLAST sequence alignment of LCTX-F2 with other homologs. Identical amino acid residues are indicated by the symbol (*). Cysteine residues are indicated by the symbol ^(#)^.

The *L. singoriensis* venom gland cDNA library comprised 3 × 10^6^ independent clones. To obtain high-quality EST sequences, clones shorter than 200 bp were excluded. A novel peptide named LCTX-F2 was identified from the cDNA library (**Figure 1A**). The open reading frame of LCTX-F2 was 315 bp long and encoded a 109-amino acid residue precursor containing a 44-residue signal peptide and a 65-residue mature peptide. A BLAST search indicated that LCTX-F2 belonged to the toxin 35 superfamily of spider toxins (**Figure 1B**), and the distribution of eight cysteines was the same as that observed in homologous toxins (LCTX-Ls1a, LCTX-Ls1d, and PT-2).

### Expression and Purification of Recombinant LCTX-F2

The LCTX-F2/pET32a (+) prokaryotic expression vector was transformed into the *E. coli* strain BL-21 (DE3). LCTX-F2 was first induced by isopropyl β-D-thiogalactoside (IPTG, 1 mM) and then purified from soluble *E. coli* lysates. LCTX-F2 was separated using a Ni^2+^ affinity column (His-binding resin) and cleaved by the TEV enzyme. After cleavage, two bands were observed in SDS–PAGE, indicating that the fusion protein had been completely cleaved (**Figure S1A**). LCTX-F2 was first isolated using a Sephadex G-75 gel filtration column (**Figure S1A**), and then further separated by RP-HPLC on a C18 column with a linear gradient from 0–50% acetonitrile in 60 min. A single peak containing LCTX-F2 was detected at the 33.7% acetonitrile gradient (**Figure S1B**). The purified LCTX-F2 was subjected to mass spectrometric (MS) analysis. LCTX-F2 was found to have a molecular weight of 7,501.50 Da (**Figure S1C**), which was 8 Da less than the theoretical molecular weight (7,509.56 Da), implying the formation of four intramolecular disulfide bridges.

### LCTX-F2 Shortened Blood Coagulation Time

**Figure 2:**
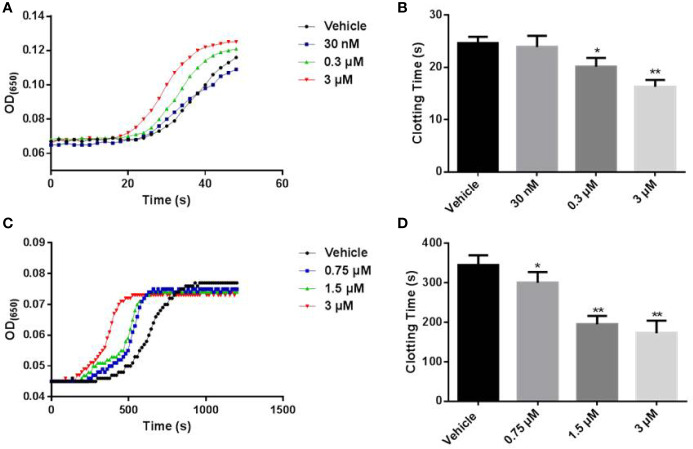
The effect of LCTX-F2 on coagulation. **(A)** The effect of LCTX-F2 on APTT. LCTX-F2 was incubated with APTT reagent (50 μl) at 37°C for 3 min. **(B)** Histogram of APTT levels. **(C)** The effect of LCTX-F2 on recalcification time. **(D)** Histogram of recalcification time with different doses of LCTX-F2. Data are presented as means ± SD of three independent experiments. *P < 0.05, **P < 0.01 compared with vehicle as determined by the two-sample Student’s *t*-test.

The effects of LCTX-F2 on blood coagulation were tested by APTT and plasma recalcification time assays. In the APTT test, 0.03, 0.3, and 3 µM LCTX-F2 significantly reduced the APTT from 24.6 s to 23.9, 20.1, and 16.3 s, respectively (**Figures 2A, B**). The recalcification time of the control was 5.8 min. After the application of 0.75, 1.5, and 3 µM LCTX-F2, the plasma recalcification time was significantly reduced to 5.0, 3.3, and 2.9 min, respectively (**Figures 2C, D**). However, in the PT test, the clotting time of the negative control was 14.3 s. After the application of 0.3 and 3 µM LCTX-F2, the plasma recalcification time was reduced to 13.7 and 13.4 s, but the reductions were not significant (**Figures S2A, B**). As previously reported, the APTT test detects intrinsic coagulation pathways, whereas the PT test detects extrinsic coagulation pathways. Therefore, our results implied that LCTX-F2 had an effect on intrinsic coagulation pathways.

### LCTX-F2 Increased Procoagulant Activity

**Figure 3:**
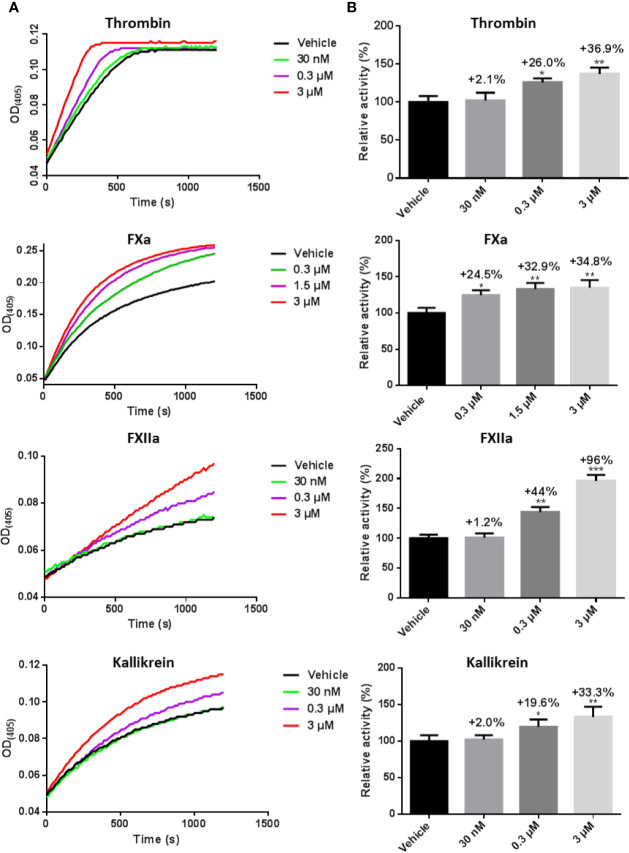
LCTX-F2 increased the activity of coagulation factors. **(A)** After incubating with 0.03, 0.3, and 3 µM LCTX-F2, the activities of thrombin, FXa, FXIIa, and kallikrein were evaluated with the corresponding chromogenic substrates for 20 min. Relative activity was calculated through enzymatic reactions. **(B)** Histogram depicting the percent increases in coagulation factor activity stimulated by LCTX-F2. Data are presented as means ± SD of three independent experiments. *P < 0.05, **P < 0.01 compared with vehicle as determined by the two-sample Student’s *t*-test.

Because we found that LCTX-F2 reduced the coagulation time, we further tested how LCTX-F2 affects coagulation factors and platelet aggregation. After LCTX-F2 application at 0.03, 0.3, and 3 µM, the relative protease activity of FXIIa was increased by 1.2%, 44.0%, and 96.0%, respectively. At 3 µM, LCTX-F2 increased the activity of thrombin, FXa, and kallikrein by approximately 36.9, 34.8, and 33.3%, respectively (**Figures 3A, B**). LCTX-F2 increased the activity of FXIIa significantly more than that of FXa and kallikrein. However, LCTX-F2 application had no effect on FVIIa, plasmin, and trypsin activity (**Figures S3A–C**). In the platelet aggregation assay, at 3 μM, no promotive effect of LCTX-F2 was observed (**Figures S3D, E**).

### Interaction Kinetics Between LCTX-F2 and Coagulation Factors

**Figure 4:**
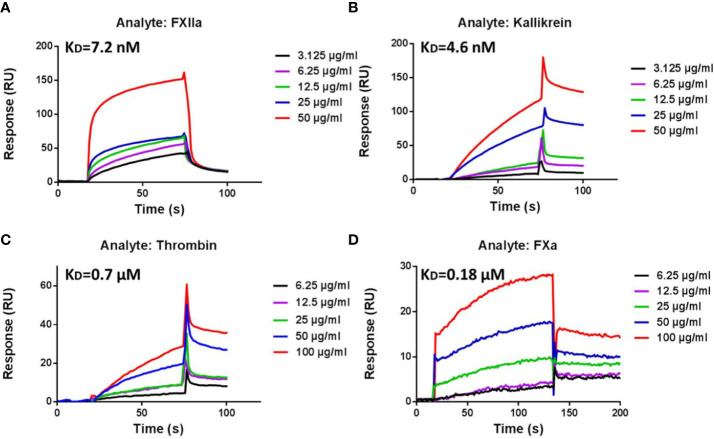
Surface plasmon resonance (SPR) sensorgrams of interactions between LCTX-F2 and coagulation factors. The concentration of LCTX-F2 to FXIIa **(A)**, kallikrein **(B)**, thrombin **(C)**, and FXa **(D)** (from bottom to top) was 0, 3.125, 6.25, 12.5, 25, 50, and 100 μg/ml, respectively. Curves were analyzed with BIAevaluation software.

As LCTX-F2 increased the activity of several coagulation factors, we tested whether LCTX-F2 directly interacts with these factors using a real-time SPR binding assay. The coagulation factors FXIIa, kallikrein, thrombin, and FXa were injected onto a chip containing immobilized LCTX-F2. Sensorgrams of the interactions between LCTX-F2 and the evaluated coagulation factors are shown in **Figure 4**. The binding affinity (*K*_D_) of LCTX-F2 for FXIIa, kallikrein, thrombin, and FXa was 7.2, 4.6, 0.7, and 0.18 µM, respectively (**Figure 4**). LCTX-F2 showed stronger affinity for FXIIa and kallikrein than for FXa and thrombin. FXa and thrombin are reported to belong to the common coagulation pathway, whereas FXIIa and kallikrein belong to the intrinsic coagulation pathway. These results indicated that LCTX-F2 promoted blood coagulation through increasing the activity of the blood coagulation factors FXIIa, kallikrein, thrombin, and FXa.

### LCTX-F2 Decreases Traumatic Hemorrhage

**Figure 5:**
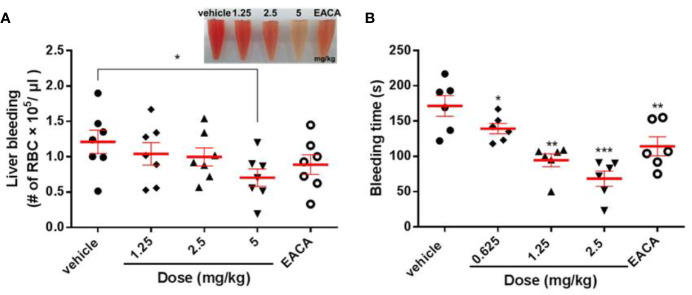
The effect of LCTX-F2 on bleeding model *in vivo*. The hemostatic effect of LCTX-F2 (0.65, 1.25, and 2.5 mg/kg) and 5 mg/kg EACA were measured in liver bleeding **(A)** and tail bleeding **(B)** mouse models. **(A)** The number of red blood cells (RBCs) was counted to determine the effect of LCTX-F2 and EACA (*n* = 7). RBCs are shown in **(A)**. **(B)** Bleeding time was determined as the time from tail transection to the moment the blood flow stopped for 120 s (*n* = 6). Data are presented as means ± SD. *P < 0.05, **P < 0.01, ***P < 0.001 compared with vehicle as determined by the two-sample Student’s *t*-test.

As shown in **Figure 5A**, the RBC count in the liver bleeding mouse model was 1.21 × 10^5^/μl. After injection with 1.25, 2.5, or 5 mg/kg LCTX-F2 and 5 mg/kg EACA, the RBC count significantly decreased to 1.04 × 10^5^, 0.99 × 10^5^, 0.70 × 10^5^, and 0.88 ×10^5^/μl, respectively (**Figure 5A**). In the tail bleeding model, a 7-mm wound was made in the mouse tail to assess homeostasis. Compared with that of vehicle, tail vein injection of LCTX-F2 at 0.625, 1.25, or 2.5 mg/kg and EACA at 5 mg/kg significantly decreased the bleeding time from 171.5 to 139.3, 94.5, 68.5, and 114.3 s, respectively (**Figure 5B**). These results suggested that, at the same dose, LCTX-F2 had a better hemostatic effect than EACA.

### LCTX-F2 Showed No Cytotoxic or Hemolytic Activities

**Figure 6:**
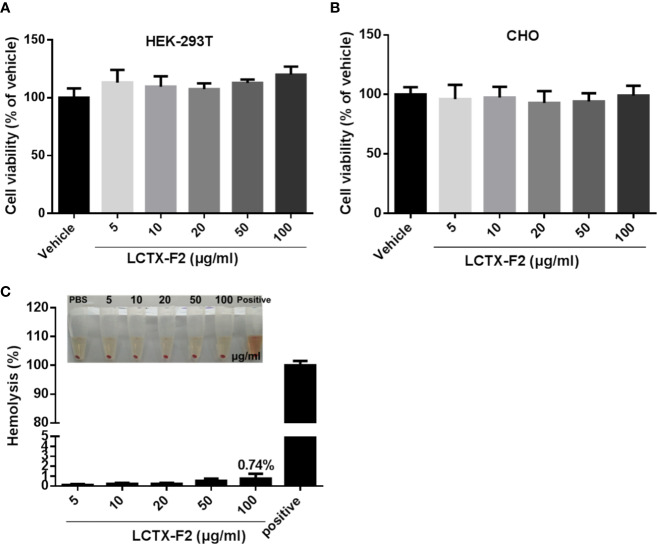
The influence of LCTX-F2 on normal cells or cell lines. LCTX-F2 did not affect the viability of HEK-293T **(A)** or CHO **(B)** cells. **(C)** Incubation with LCTX-F2 (0–100 µg/ml) did not elicit hemolytic effects against red blood cells.

Incubation with LCTX-F2 at concentrations ranging from 0 to 100 µg/ml for 24 h did not inhibit the viability of HEK-293T or CHO cells (**Figures 6A, B**). In the hemolysis assay (**Figure 6C**), LCTX-F2 did not exhibit hemolytic activity, even at the concentration of 100 µg/ml.

## Discussion

Peptides derived from spider venom are highly diverse, and include metalloproteinases, serine proteinases, hyaluronidases, serine/cysteine proteinase inhibitors, alkaline phosphatases, ATPases, and insecticidal toxins (Machado et al., 2005; Chaim et al., 2011; Jin et al., 2017). LCTX-F2 consists of 65-amino acid residues, including four intramolecular disulfide bridges. The BLAST results showed that LCTX-F2 belongs to the toxin 35 family and shares high sequence identity with several known peptide toxins from wolf spiders. The number and positions of cysteines are highly conversed in the toxin 35 family, implying that LCTX-F2 also contains an ICK motif toxin (Zhang et al., 2010). The function of spider toxin 35 family members is not known, except that of purotoxin-2, which has been shown to act on ion channels (Liang, 2004; Liang, 2008; Kabanova et al., 2012).

In this study, LCTX-F2 significantly reduced the plasma recalcification time and APTT in a dose-dependent manner. Results of the SPR assay indicated that LCTX-F2 is a novel agonist that has affinity for, and directly interacts with, FXIIa, kallikrein, thrombin, and FXa. Moreover, LCTX-F2 did not enhance the activity of plasmin, trypsin, or FVIIa, or reduce platelet aggregation time. Evaluation of the effect of LCTX-F2 on traumatic hemorrhage in liver bleeding and tail bleeding mouse models showed that LCTX-F2 exhibited a more significant procoagulant effect than EACA at the same dosage. Furthermore, cytotoxicity and hemolysis assays indicated that LCTX-F2 exerted no cytotoxic or hemolytic effects on normal cells.

**Figure 7:**
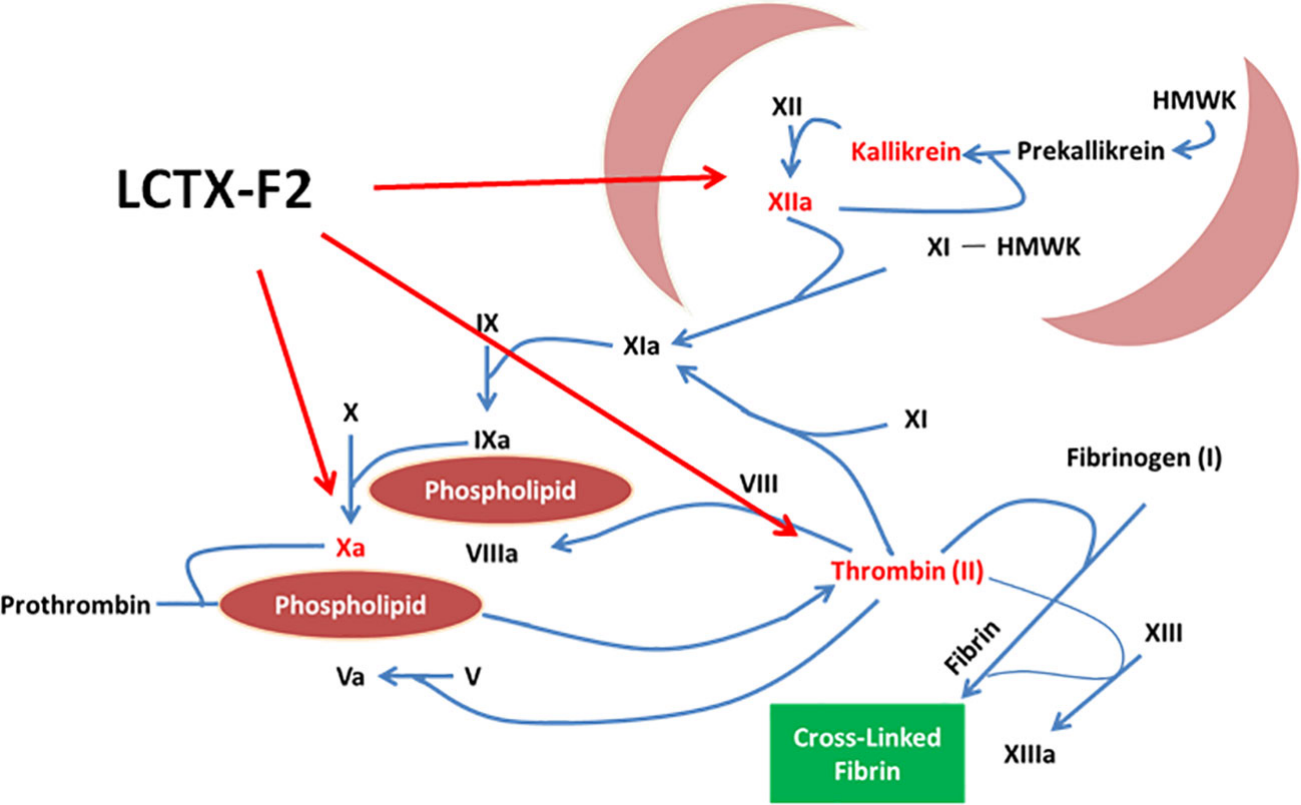
LCTX-F2 promoted blood coagulation by increasing the activity of intrinsic blood coagulation factors, including FXIIa, kallikrein, thrombin, and FXa.

Hemostasis is a complex physiological process encompassing initiation, amplification, propagation, and stabilization (Macfarlane, 1964). Hemostasis has also been traditionally classified into intrinsic and extrinsic pathways, and a further pathway common to both (Grover and Mackman, 2019). The intrinsic pathway is triggered by activation of FXII, followed by the sequential proteolytic activation of FXI and FIX. Formation of the intrinsic Xase (a complex of FVIIIa and FIXa) leads to the formation of the intrinsic FXa; in turn, FXa and FVa form the prothrombinase complex that catalyzes the cleavage of prothrombin (FII) to thrombin (FIIa). Thrombin is the terminal coagulation factor that cleaves soluble fibrinogen to insoluble fibrin. Procoagulant drugs such as factor-Xa like proteases, TXA, EACA, and kaolin have been used in clinical treatment. TXA and EACA, two antifibrinolytic agents, competitively inhibited the conversion of plasminogen to plasmin. Kaolin as an emergency trauma hemostatic agent could activate FXII proenzyme into FXIIa. Factor X-like proteases from the venom of snakes (Siigur et al., 2001; Larreche et al., 2008; Sajevic et al., 2011; Khan and Al-Saleh, 2015) are either metalloproteases or serine proteases and could activate prothrombin to thrombin. In this study, LCTX-F2 promoted blood coagulation by increasing the activity of intrinsic blood coagulation factors, including FXIIa, kallikrein, thrombin, and FXa (**Figure 7**).To the best of our knowledge, LCTX-F2 is the first peptide activates coagulation factors and shortens blood coagulation time using mechanisms different from those previously reported for other proteins or drugs.

In summary, we identified LCTX-F2 as the first procoagulant peptide in spider venom. LCTX-F2 promoted coagulation by increasing the activity of blood coagulation factors. LCTX-F2 showed a better procoagulant activity than EACA and exhibited no cytotoxic or hemolytic activity against normal cells. LCTX-F2 may be a good candidate and/or template for the development of procoagulant drugs.

## Data Availability Statement

The LCTX-F2 sequence has been uploaded to GenBank of NCBI under the accession number BankIt 2321489 MT178457.

## Ethics Statement

The animal study was reviewed and approved by the Animal Care and Use Committee at Kunming Institute of Zoology, Chinese Academy of Sciences (SMKX-20170210).

## Author Contributions

PL, ZZ, QL, and EM performed the experiments and analyzed the data. RL and MR conceived and supervised the project. PL, JM, RL, and MR prepared the manuscript. All authors contributed to the article and approved the submitted version.

## Funding

This research was funded by the Education Department of Hunan Province (19A321), the National Natural Science Foundation of China (31971190, 81703400, 31801975), Huxiang High Level Talent Gathering Project (2018RS3071), Chinese Academy of Sciences (SAJC201606, ZSTH-034), and Science and Technology Department of Yunnan Province (2019ZF003, 2019FB040 and 2018ZF001).

## Conflict of Interest

The authors declare that the research was conducted in the absence of any commercial or financial relationships that could be construed as a potential conflict of interest.

## Abbreviations

APTT, activated partial thromboplastin time; PT, prothrombin time; FXIIa, activated factor XII; FXa, activated factor X; rFVIIa, recombinant activated factor VII; EACA, epsilon aminocaproic acid; TXA, tranexamic acid; HEK-293T, Human embryonic kidney 293T; CHO, Chinese Hamster Ovary 1; PBS, phosphate-buffered saline; PPP, platelet-poor plasma; IPTG, Isopropyl β-D-thiogalactoside; RBC, red blood cell.
