# Supplementary material for: LCTX-F2, a Novel Potentiator of Coagulation Factors From the Spider Venom of Lycosa singoriensis
Source: Front Pharmacol. 2020 Jun 16;11:896. doi: 10.3389/fphar.2020.00896 (PMC7308506; doi:10.3389/fphar.2020.00896)
Supplement: Supplementary file 1 [file DataSheet_1.docx]

Supplementary Material

# Supplementary Figures

## Figure S1


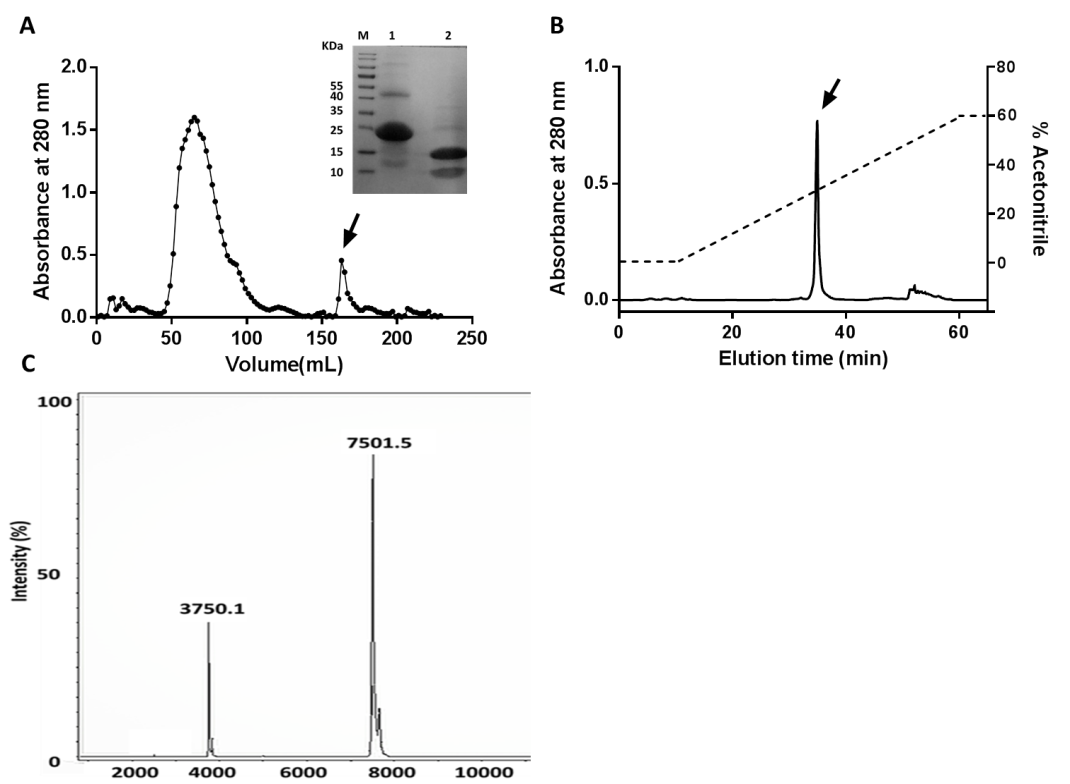


**Supplementary Figure 1.** Recombinant expression and purification of LCTX-F2. (A) Recombinant LCTX-F2 was purified by Sephadex G-75 gel filtration. SDS-PAGE of samples after TEV enzyme cleavage (inserted in A): collected fusion proteins were purified with affinity (line 1), recombinant LCTX-F2 was purified by a Sephadex G-50 (line 2) after TEV enzyme cleavage. (B) Recombinant LCTX-F2 was purified by C18 RP‐HPLC. The elution was performed with the gradients of acetonitrile in 0.1% (v/v) trifluoroacetic acid in water at a flow rate of 1 mL/min. During the time from 10 to 60 min, the gradient of acetonitrile was increased from 10% to 50%. (C) The molecular weight of recombinant LCTX-F2 was determined by MALDI-TOF mass spectrometry.

## Figure S2


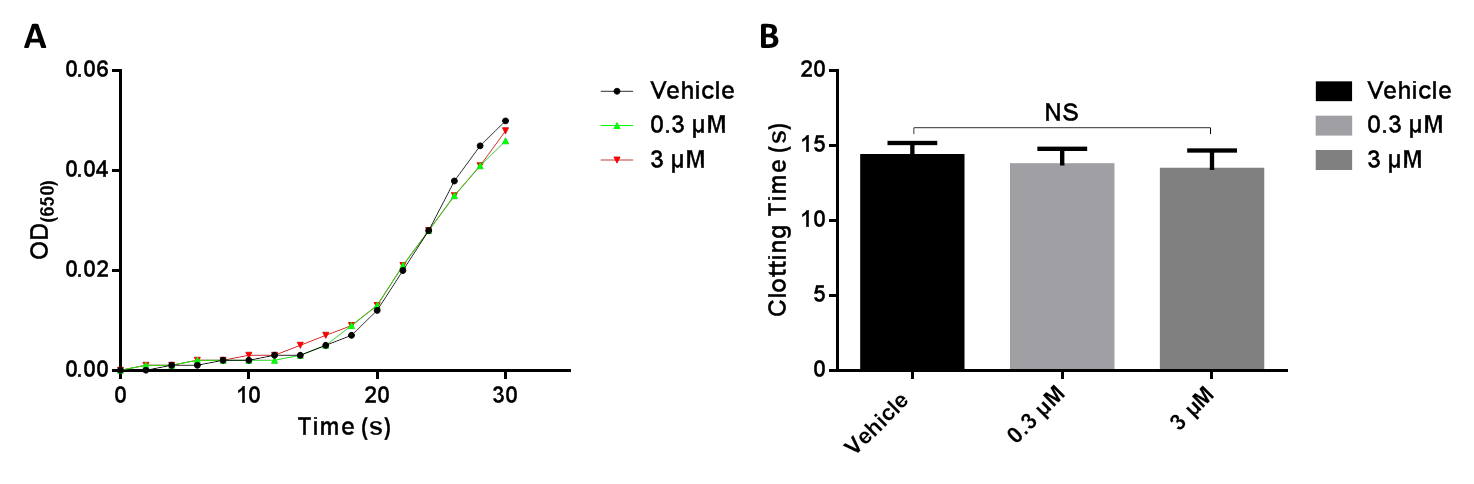


**Supplementary Figure 2.** Effect of LCTX-F2 on coagulation. (A) Effect of LCTX-F2 on PT test. PT reagent was incubated with LCTX-F2 at different dose. (B) The clotting time of PT test was transformed into [histogram](file:///D:\Program%20Files\Youdao\Dict\7.2.0.0703\resultui\dict\?keyword=histogram). Data were presented as mean ± SD of three independent experiments. *P< 0.05, ** P< 0.01, compared with vehicle, analyzed by student’s t test for two-sample comparison.

**Figure S3**


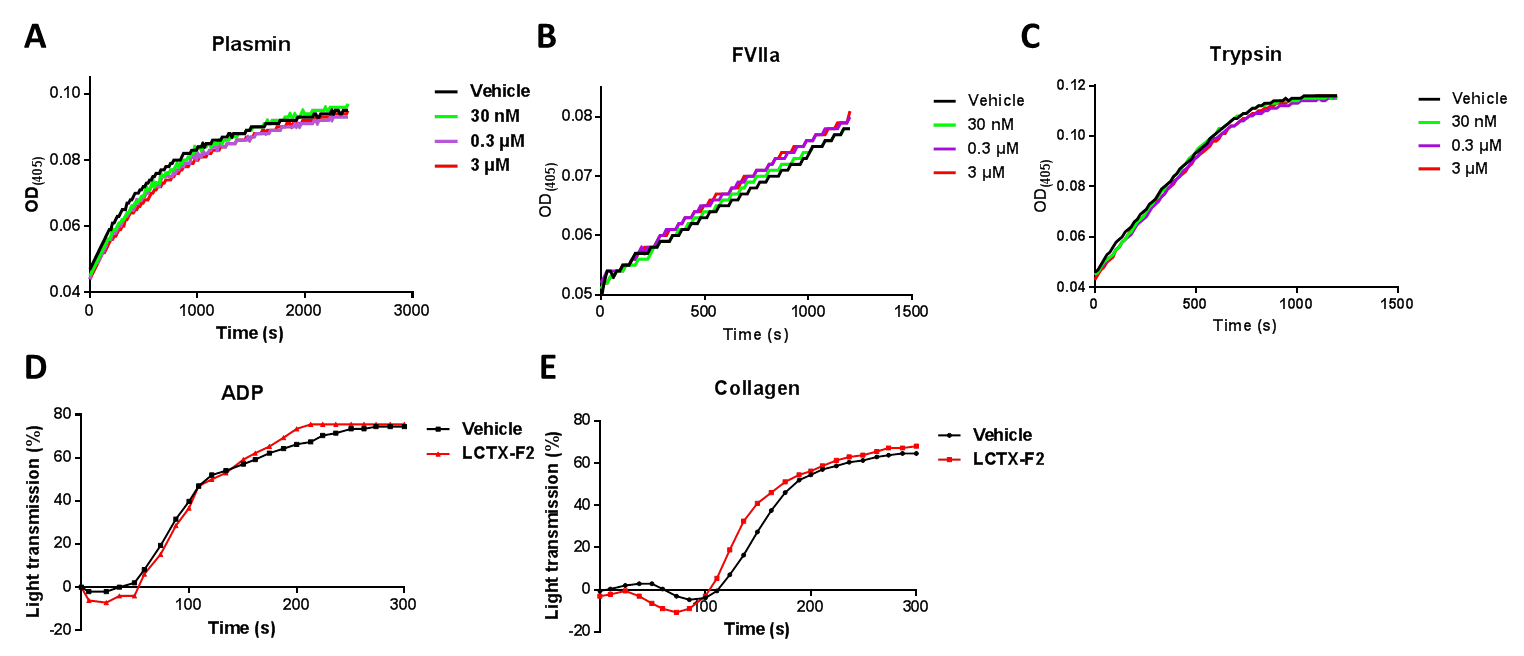


**Supplementary Figure 3.** LCTX-F2 did not have promoting function to some other protease involving plasmin (A), FVIIa (B), trypsin (C) and platelet aggregation (D, E).
